# Job loss during pregnancy and the risk of miscarriage and stillbirth

**DOI:** 10.1093/humrep/dead183

**Published:** 2023-09-27

**Authors:** Alessandro Di Nallo, Selin Köksal

**Affiliations:** Dondena Centre for Research on Social Dynamics and Public Policy, Bocconi University, Milan, Italy; Institute for Social and Economic Research, University of Essex, Colchester, UK

**Keywords:** job loss, stillbirth, miscarriage, adverse pregnancy outcome, psycho-social stress, Understanding Society, UK

## Abstract

**STUDY QUESTION:**

Does the exposure to job loss during pregnancy increase the risk of miscarriage or stillbirth?

**SUMMARY ANSWER:**

The experience of own or partner’s job loss during the pregnancy is associated with an increased risk of miscarriageand stillbirth.

**WHAT IS KNOWN ALREADY:**

Prior research on the psycho-social aspect of pregnancy loss has investigated the contextual and the individual-level stressors. At the contextual level, natural disasters, air pollution, and economic downturns are associated with higher risk of pregnancy loss. At the individual level, intense working schedules and financial strain are linked with increased risk of pregnancy loss both at early and later stages of the gestation.

**STUDY DESIGN, SIZE, DURATION:**

This work draws on high-quality individual data of ‘Understanding Society’, a longitudinal survey that has interviewed a representative sample of households living in the UK annually since 2009. Approximately 40 000 households were recruited. The analyses use all the available survey waves (1–12, 2009–2022).

**PARTICIPANTS/MATERIALS, SETTING, METHODS:**

The final sample consisted of 8142 pregnancy episodes that contain complete informationon pregnancy outcome and date of conception. Ongoing pregnancies at the time of the interview were excluded from the final sample. The outcome variable indicated whether a pregnancy resulted in a live birth or a pregnancy loss whereas the exposure variable identified the women’s or their partner’s job loss because of redundancy or a dismissal. Logistic regression models were employed to estimate the relation between job loss during pregnancy and pregnancy loss. The models were adjusted for an array of socio-demographic and economic characteristics following a stepwise approach. Several sensitivity analyses complemented the main findings.

**MAIN RESULTS AND THE ROLE OF CHANCE:**

Baseline models controlling for women’s demographic background and prior experience of miscarriage estimated an increased risk of pregnancy loss when women were exposed to their own or their partner’s job loss during their pregnancy (odds ratio (OR) = 1.99, 95% CI: 1.32, 2.99). When the models were adjusted for all socio-economic and partnership-related covariates the association remained robust (OR = 1.81, 95% CI: 1.20, 2.73).

**LIMITATIONS, REASONS FOR CAUTION:**

First, the pregnancy outcome and the date of conception were self-reported and may besubjected to recall and social desirability bias. Second, although we adjusted for an array socio-demographic characteristics and self-reported health, other contextual factors might be correlated with both job loss and pregnancy loss. Third, owing to the limited sample size, we could not assess if the main finding holds across different socio-economic strata.

**WIDER IMPLICATIONS OF THE FINDINGS:**

By showing that exposure to a job loss during pregnancy increases the risk of miscarriage and stillbirth, we underline the relevance of pregnancy loss as a preventable public health matter. This result also calls for policy designthat enhances labour market protection and social security buffers for pregnant women and their partners.

**STUDY FUNDING/COMPETING INTERESTS:**

The authors received the following financial support for the research, authorship, and/or publication of this article: H2020 Excellent Science, H2020 European Research Council, Grant/Award Number: 694262 (project DisCont—Discontinuities in Household and Family Formation) and the Economic and Social Research Centre on Micro-Social Change (MiSoC). There are no conflicts of interest to declare.

## Introduction

Pregnancy loss is a collective term used to identify a pregnancy that does not result in a live birth (Flenady *etal.*, 2016; Quenby *etal.*, 2021), and might occur any time during the course of pregnancy. According to National Health Service (NHS) in the UK, it is termed ‘miscarriage’ when it occurs before the 24^th^ week of pregnancy, and ‘stillbirth’ thereafter (Quenby *etal.*, 2021). In high-income countries, 11–21% of clinically detected pregnancies result in miscarriage (Bruckner *etal.*, 2016) while ∼0.3–0.5% of pregnancy losses occur as stillbirths (Flenady *etal.*, 2016).

Despite its social and economic relevance, pregnancy loss is still a rarely discussed public health matter in private and public spheres owing to the stigma and shame attached to the adverse reproductive experiences (Bellhouse *etal.*, 2018). Pregnancy losses are consequential for women and their partners’ health and well-being as they are associated with an increased risk of long-term depression and anxiety-related symptoms (Blackmore *etal.*, 2011; Hogue, 2016). They are also costly for healthcare systems, as the yearly economic cost of miscarriages is estimated at £471 million in the UK (Quenby *etal.*, 2021). Despite the improvements in maternal and neonatal mortality rates, the prevalence of stillbirths has remained relatively stable (Bruckner *etal.*, 2016; Hogue, 2016). In the UK in the last 5 years, the number of stillbirths has steadily exceeded the number of liveborn infants who die before their first birthday (Office for National Statistics, 2020). Our knowledge on the prevalence of miscarriage across different cohorts and periods remains rather limited owing to the scarcity of official statistics.

Prior research has uncovered several antecedents of pregnancy loss such as advanced maternal age, chromosomal abnormalities, lifestyle factors, and genetic propensity (Dimitriadis *etal.*, 2020). Moreover, exposure to environmental stressors during the pregnancy, such as natural disasters (Torche, 2011), air pollution (Kioumourtzoglou *etal.*, 2019), and economic downturns (Bruckner *etal.*, 2016), may put pregnancies at risk as well. Ecological evidence shows that the probability of reporting a pregnancy loss increases during periods of rising unemployment (De Cao *etal.*, 2022), while individual-level studies found that other stressful life events, including financial strain as well as intense work schedules, are linked with higher risk of pregnancy loss both at early and late stages of pregnancy (Neugebauer *etal.*, 1996; Whelan *etal.*, 2007; Hogue *etal.*, 2013). A job loss for women or for their partners’ is generally considered an external source of stress and discomfort as it indicates involuntary job termination that occurs when workers are laid off (Brand, 2015). The studies focusing on individual labour market shocks have shown that job loss is negatively associated with a wide array of children’s perinatal outcomes, such as birthweight, gestational length, and foetal growth (Dooley and Prause, 2005; Lindo, 2011; Gailey *etal.*, 2022a, b). This could also suggest that the risk of pregnancy loss might be associated with the exposure to an involuntary job loss, although no evidence has been provided so far. Ajob loss could be associated with increased level of stress hormones (Sumner and Gallagher, 2017), reduction in financial and time resources (Brand, 2015), frustration and, eventually, health-harming behaviour (e.g. tobacco and substance use, unhealthy eating) during the pregnancy period (De Cao *etal.*, 2022), all of which might jeopardize the course of pregnancy (Scharber, 2014).

To date, however, no study that we are aware of has examined whether a job loss experienced by a woman, or her partner, during the pregnancy is associated with higher risk of pregnancy loss. This constitutes a gap in knowledge as job loss is a disruptive life event and it is a source of discomfort, which has far-reaching health consequences for women and their kin (Brand, 2015; Aquino *etal.*, 2022). We hypothesize that women who experience their own or their partners’ job displacement are under higher risk of experiencing a pregnancy loss, namely miscarriage or stillbirth, compared to their counterparts who have not been exposed to a job loss during their pregnancy.

Our study contributes to the literature by providing original evidence on the association between job loss and pregnancy disruption. Using granular information on pregnancies together with women and their partners’ employment histories, we can estimate whether the risk of pregnancy loss changes by the exposure to women’s or partners’ job loss during the pregnancy. Further, we include an array of socio-demographic characteristics of women and their partners to examine whether the association remains robust. In further sensitivity analyses, we use different definitions of pregnancy loss, include other indicators of ‘job separation’, address the timing of job loss with respect to the conception, introduce a categorical variable that separates women’s job loss from their partner’s as the new exposure variable, and we re-specify the models without including the previous experience of miscarriage. We use data from sweeps 1–12 of ‘Understanding Society’, the UK Household Longitudinal Study (UKHLS), which provides fine-grained information on pregnancy histories and labour market events on a monthly basis along with individuals’ socio-demographic characteristics.

## Materials and methods

### Data and variables

The UKHLS is a longitudinal survey of 40 000 households living in the UK that was conducted annually from 2009 to 2022. The survey employs address-based sampling and stratified sampling techniques to ensure representativeness from different geographic areas and population groups. It collects information on many life domains, including fertility, health, and family life from all household members aged 16 years or older. In each yearly wave, the women who experience a pregnancy can report date of conception (month and year), gestational length (months), cause of interruption, and delivery outcome (Supplementary Fig. S1). Also, the survey provides information on workplace changes, such as the type of job interruption (e.g. redundancy, dismissal, contract end, etc.) and its date (month and year), on a yearly basis. Thus, in each wave it is possible to detect if, and when, any episodes of pregnancy disruption and job loss occurred. As the data are structured via annual interview, it minimizes the risk of recall bias (Supplementary Table S1). The overall proportion of sampled households who took part in the survey at Wave 1 was 57.3% in the general population sample, in line with comparable surveys (Benzeval *etal.*, 2020). The attrition ranges from 25.9% between Waves 1 and 2–2.0% between Waves 11 and 12. We construct a pregnancy-level dataset, in which each woman can report more than one pregnancy during the observation period. Our final sample size consists of 8142 episodes of pregnancies (for 4942 women and associated partners—if any—at a given time). We excluded the ongoing pregnancies at the last wave and the pregnancies reported by women who dropped out of the survey some time over the 12 waves under consideration, and before any pregnancy outcome was reported (N* *=* *467); conceptions with no reported date (N* *=* *319); and pregnancies with unknown outcome (N* *=* *32, Supplementary Fig. S2).

For each self-detected pregnancy, we identify whether it results in a live birth or not. The outcome variable is a dichotomous variable equal to 1 if the respondent experienced a miscarriage or a stillbirth, and 0 otherwise. We exclude pregnancy termination (abortion) cases from the main analysis, as the focus of this study is primarily on pregnancy loss, defined by miscarriage or stillbirth.

Miscarriages constitute about 12% (N* *=* *982, Table 1) of the reported conceptions, in line with the estimates of clinically detected spontaneous losses (Bruckner *etal.*, 2016). As the majority of pregnancies do not survive within the first month and a large proportion of pregnancies losses are undetected, the reported miscarriages may be underestimated compared to all actual pregnancy losses (Dimitriadis *etal.*, 2020). Reassuringly, previous research did not find any evidence that reports of pregnancy outcomes were being influenced by the episodes of economic hardship (Desai *etal.*, 2021). Further, the main analysis measuring the association between job loss and pregnancy loss would potentially produce a conservative estimate as the early miscarriages are more likely to go unnoticed or to be unreported.

**Table 1.** Summary statistics of the outcome variables and covariates by exposure to a job loss during pregnancy.

	Job loss		No job loss		Total	
	N = 136		N = 8006		N = 8142	
	N	%	N	%	N	%
*Pregnancy loss*	33	24.3	952	11.9	985	11.0
Miscarriage	32	23.5	915	10.4	947	11.6
Stillbirth	1	0.7	37	0.5	38	0.5
	
*Woman's characteristics*	%		%		%	
	
Age at conception (years, mean/SE)	31.23 (5.97)		30.47 (5.87)		30.49 (5.87)	
Married or cohabiting at conception	86.0		79.3		79.4	
Has a university degree	45.6		36.1		36.3	
White ethnic origin	78.7		76.7		76.7	
Parents were upper-middle class	25.0		25.3		25.3	
Has children	30.1		30.7		30.7	
Had at least one prior miscarriage	11.0		4.8		4.9	
Health reported as ‘fair’	10.3		7.3		7.3	
Health reported as ‘poor’	1.9		1.7		1.7	
*Partner's characteristics*						
Age at conception (years, mean/SE)	34.23 (6.18)		34.02 (6.43)		34.02 (6.43)	
Job class (intermediate)	11.8		10.3		10.3	
Job class (manager and professional)	30.9		24.8		24.6	
*Monthly household income in GB* pounds (mean/SE)	2644 (5090)		2786 (5402)		2784 (5371)	

Source: Understanding Society, UK (2009–2022).

Stillbirths represent 0.47% of the conceptions (N* *=* *38, Table 1), in keeping with the official statistics in the UK, as stillbirths have ranged between 0.36 and 0.45 per hundred pregnancies during the 2009–2022 period (Office for National Statistics, 2020).

We exclude pregnancy termination (abortion) cases from the main analysis, as the focus of this study is primarily on involuntary pregnancy loss. Voluntary terminations constitute ∼18–25% of all detectable pregnancies in high-income countries (Sedgh *etal.*, 2012) and are also found associated with involuntary pregnancy losses (Catalano *etal.*, 2016). According to the official statistics, the age-standardized abortion rate is 1.8 per hundred pregnancies in the UK (Department of Health and Social Care, 2020), while they represent 2.3% in our sample (N* *=* *191).

The primary exposure variable identifies if an involuntary job loss experienced by women, or their partners, occurs after the date of conception or before the delivery. Unlike other similar studies (Scharber, 2014), we can distinguish *involuntary* job losses, which occur because of dismissal or redundancy. We thus follow previous literature that has already addressed this source of involuntary job loss in the UK data. One main advantage of UKHLS is that women report the length of gestation that ends prematurely in a non-live birth. This piece of information can be combined with the timing of a job loss, also expressed at the monthly level, so it is possible to detect if the exposure to job loss occurred during the pregnancy. For instance, if a pregnancy loss occurs within the third month from the conception, and job loss falls within the third month, the binary indicator equals 1. Conversely, if a pregnancy loss occurs within the third month from the conception, and the displacement happens 6 months after the conception date, the binary indicator is 0. If conception and job loss occur in the same month, we consider the couple to be exposed to the shock. Nevertheless, there are no cases of a non-live birth and a job loss happening in the same month (Supplementary Tables S2 and S3). In additional tests, we also specify the gender of the partner hit by the job displacement (see Sensitivity analyses section).

All the other causes of job end, such as contract termination, illness, parental leave, retirement, or unspecified reasons, are *not* considered involuntary (Brand, 2015) and their binary indicator is 0. These episodes are considered for alternative specifications of the primary exposure variable in the Sensitivity analyses section.

We include a set of covariates to account for several confounding factors (Supplementary Fig. S3). We control for demographic characteristics: women’s age at the time of the interview (in 3-year groups), ethnicity, parents’ highest class when the women were 16 years old. We also adjust for any previously reported miscarriages or stillbirths, in line with prior research on pregnancy loss (Dimitriadis *etal.*, 2020). We then include a proxy of women’s socio-economic status (SES), such as the highest educational attainment (No qualification, Other qualification, GCSE, etc., A level etc., Other higher degree, Degree), a measure of self-reported health (five categories from ‘Poor’ to ‘Excellent’), and maternal status (if they have a child or not). Finally, we adjust for the marital status at conception (cohabiting, married, single); partner’s age and job position (NSSeC-3, a British scale), if any co-residential partner is reported, and household income (Supplementary Table S4). All these covariates are lagged with respect to the conception date. In all models, temporal patterns in pregnancy loss (e.g. seasonality, trend) are captured by year and month fixed effects. More details about variables’ definitions and operationalizations are provided (Supplementary Table S5).

### Statistical analysis

We employ a logistic regression to estimate the association between job loss and the risk of pregnancy loss. First, we estimate a baseline model predicting the risk of non-live birth including the primary exposure variable and covariates for women’s demographic characteristics and background—age, ethnicity, and parents’ social class—the experience of a prior pregnancy loss, and year and month fixed effects. Second, we add women’s educational attainment, marital status, and subjective health. Finally, we add partner’s SES, if a partner is reported, and household income. Heteroskedasticity-robust Ses are clustered at the individual level to adjust for non-independence of conceptions within women over time.

## Results

### Main findings

Our findings show that women who are exposed to their own or their partner’s job loss during the pregnancy have increased risk of pregnancy loss (Table 2) as compared to the ones who were not (odds ratio (OR) =1.985, 95% CI: (1.319–2.987)). The results remain robust as new covariates are added to the baseline specification, which only features the primary exposure variable, women’s age, ethnicity and parents’ SES, the month and year fixed effects, and self-reported prior experience of miscarriage (OR = 1.831, 95% CI: (1.220–2.748)). In Model 3, which includes all the covariates, the odds of experiencing a pregnancy loss when women were exposed to their own or their partner’s job loss are estimated to be 1.812 (CI: (1.204–2.728)) compared to women who have not experienced any job loss (Supplementary Table S6).

**Table 2.** Results from logistic models regressing involuntary job loss on pregnancy outcomes.

Parameter	Odds ratio	95% CI	Observations
*Ref*: no job loss			
Job loss			
*Model 1*^a^	1.985	1.319, 2.987	8142
*Model 2*^b^	1.831	1.220, 2.748	8142
*Model 3*^c^	1.812	1.204, 2.728	8142

Source: Understanding Society, UK (2009–2022).

a Model 1 includes: age (linear, quadratic, cubic), ethnicity, parents’ highest social class, previous experience of miscarriage, year, and month fixed effects.

b Model 2 includes: woman’s educational attainment, partnership status, self-reported general health, in addition to the confounders of Model 1.

c Model 3 includes: partners’ educational attainment and NS-SeC3 job status, and household income (in logarithm), in addition to the confounders of Model 2.

Full estimates are displayed in Supplementary Table S6.

**Figure 1.**
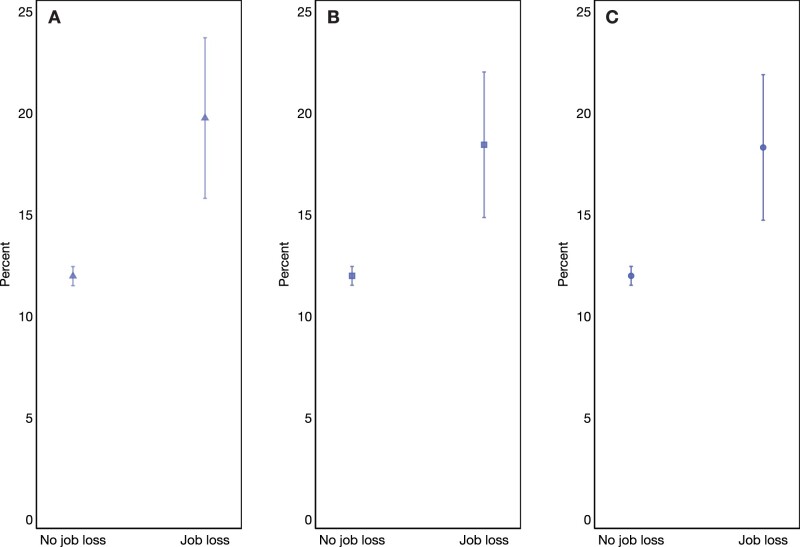
**Predicted probability for risk of miscarriage or stillbirth for pregnancies affected and not affected by woman’s or her partner’s job loss.** (**A–C**) The results obtained from Model 1 (baseline + woman’s demographic characteristics and prior miscarriage), Model 2 (Model 1 + woman’s SES and partner’s characteristics), and Model 3 (Model 2 + partner’s SES and household income), respectively. The 95% CIs are shown. Source: Understanding Society, UK (2009–2022). SES: socio-economic status.

Figure 1 shows the predicted probability of pregnancy loss derived from the three models. The estimated probability of pregnancy disruption in the absence of job loss is ∼12% in all specifications, in line with prior estimates (Bruckner *etal.*, 2016; Quenby *etal.*, 2021), which confirms the reliable self-reporting of recognized conceptions. Experiencing job loss during the pregnancy increases the probability of pregnancy loss to ∼20% (Fig. 1A, Model 1) and 18% (Fig. 1C, Model 3).

### Sensitivity analyses

We performed several sensitivity analyses, which are presented in Supplementary Tables S7, S8, S9, S10, S11, and S12. First, we used two alternative outcomes: miscarriage and non-live birth (aterm encompassing miscarriage, stillbirth, and abortion), which are more and less restrictive definitions, respectively, of the outcome used in the main analysis. These findings confirm the accuracy of the operationalization used in the main analysis (Supplementary Tables S7 and S8).

Second, we replicated our analyses with a categorical exposure variable: involuntary job loss (as in the main analysis); an *anticipated* or non-involuntary contract termination (‘temporary job end’, ‘other’, and ‘non-specified’); a voluntary exit from the labour market (e.g. illness, unspecified reason, etc.); no labour market change. We tested whether other causes of job termination gauge the condition of uncertainty and are associated with an increase in the risk of pregnancy disruption. The analysis reveals that *only* involuntary job losses—the primary exposure variable—are associated with an increase in the risk of pregnancy loss (*P* < 0.01, Supplementary Table S9).

Third, we addressed the *timing* of job loss with respect to the *timing* of conception in more detail. We replicated the main analysis with a categorical exposure variable: a job loss *preceding* conception by 12–1 month; exposure to job loss during the pregnancy (as in the main analysis); a job loss between the gestation end and 12 months thereafter; all the residual cases (a job loss in another period, or no job loss). Thus, this specification accounts for pregnancy losses that occurred prior to the job loss. We acknowledge that couples who experience a job loss and subsequently choose to conceive might still suffer stress-related consequences. A job loss on this margin does not affect a pregnancy disruption, but it may change pre-pregnancy health behaviours that affect a conception, namely sexual activity and/or contraception use. The findings confirm that only job loss during the gestation is associated with pregnancy disruption, while *pre-conception* job loss is not linked with adverse outcomes (Supplementary Table S10).

Fourth, we conducted a supplementary analysis by examining the impact of job loss on women and their partners separately. This approach allowed us to explore the potential differences in outcomes based on gender. Women’s job loss might be more consequential in increasing the risk of miscarriage owing to the direct impact of stress and uncertainty associated with losing their jobs. Conversely, favourable circumstances for a healthy pregnancy could arise, such as the ability to avoid potentially stressful work environments and allocate more time to personal care and rest. This situation may be particularly applicable to the numerous female part-time workers in the UK labour market, who may experience relatively lower economic losses. Furthermore, previous research has suggested that a male partner’s job loss can be considered as a more exogenous source of job-related stress compared to the female partner’s job loss because it is more likely to be independent of the course of pregnancy. In contrast, women’s labour market decisions may be influenced by their pregnancy, thereby raising concerns of reverse causality (Gailey *etal.*, 2022a). In the UK, the Equality Act 2010 protects pregnant women against discrimination in the workplace. According to this legislation, employees must communicate their pregnancy to their employer at least 15 weeks before the delivery is due. To the extent that these protection policies are implemented thoroughly, they can effectively resolve the reverse causality issues. At the same time, it can give rise to selection bias as we might systematically select women who communicate their pregnancy relatively late. To address these endogeneity concerns, the primary exposure variable is replaced with a categorical variable such as: woman’s job loss; partner’s (if any) job loss; no job loss (as in the main analysis). The results of this analyses revealed that both women’s and their partners’ job loss are associated with increased risk of pregnancy loss (Supplementary Table S11).

Fifth, we estimated additional models without controlling for previous non-live births. By controlling for a previous miscarriage, we intended to control for women’s predisposition for involuntary pregnancy interruptions, as well as any other stressful periods that might have heightened their risk of pregnancy loss. Women who experienced a pregnancy loss in the past might be less willing to seek a new pregnancy. A miscarriage may also change women’s (and partners’) lifestyle, including the work conditions. It is possible that women who have experienced a pregnancy loss may shift from full– to part-time or self-select into more stable jobs. To this end, the inclusion of the variable ‘previous miscarriage’ may capture not only women’s biological predisposition to experience a pregnancy loss, but also changes in their behaviour to minimize future risk of involuntary pregnancy terminations. The results of this sensitivity test showed greater odd ratios in magnitude of the main exposure variable as compared to the main analysis (Supplementary Table S12).

## Discussion

Among the pregnancies that are clinically detected, 10–21% are spontaneously lost. Besides the biological factors, job loss is one of the possible social causes of adverse pregnancy outcomes. Previous studies have shown that economic downturns, stressful life events and intense working hours could increase the risk of pregnancy loss (Neugebauer *etal.*, 1996; Whelan *etal.*, 2007; Bruckner *etal.*, 2016). Yet, whether a job loss disrupts the course of pregnancy remains an understudied question. The existing evidence is provided through ecological studies (Bruckner *etal.*, 2016), in which the sample is based on a heterogeneous population of prospective parents, most of which do not lose a job. This limitation leaves open the question as to whether involuntary job loss in a family per se—and not the economic cycle or other events—influences the course of a pregnancy (Hogue, 2016).

This study shows an increase in the probability of pregnancy loss for pregnancies that are exposed to women’s or their partners’ job loss, using a population-based survey in the UK. The findings are consistent with the hypothesis that the combination of physiological, psycho-social, and economic hardship experienced during a pregnancy is likely to impair its continuity. Pregnancies could be at risk via one or more of these mechanisms. First, the physiological response to a source of stress triggers the production of corticotrophin-releasing hormone (CRH), adrenocorticotrophic hormone and cortisol (Nepomnaschy *etal.*, 2006). These hormones are found to increase the risk of miscarriage (Nepomnaschy *etal.*, 2006), while CRH could lead to uterine contractions and to premature delivery, which is a risk factor for stillbirth (Gravett *etal.*, 2010). Second, the reduction in the available income could restrict access and compliance to prenatal care (Geiger *etal.*, 2021). Therefore, at-risk pregnancies could be discovered late or be undetected, thus increasing the risk of pregnancy disruption or jeopardizing the health of the foetus (Gravett *etal.*, 2010). Third, the emotional discomfort of job loss could give rise to health-harming behaviours during pregnancy, such as the use of toxic substances, smoking or unhealthy eating (Hobel *etal.*, 2008; De Cao *etal.*, 2022). Our evidence is in line with research showing how psychological shocks during gestation are associated with adverse pregnancy outcomes (Scharber, 2014; De Cao *etal.*, 2022).

Further, the exposure to an anticipated but *not involuntary* source of job interruption—which we tested in the sensitivity analyses—does not produce the same results as an involuntary job loss. Although we cannot untangle the mechanisms, we can hypothesize that not only the financial uncertainty but also the reaction to stress and frustration, compatible with an involuntary but not with an anticipated job loss, seems to be at least equally harmful (Carlson, 2015; Almond *etal.*, 2018).

The identification strategy clearly distinguished job loss occurring before conception from that occurring after conception. However, a job loss could be a marker of pre-existing economic disadvantage and stress, which might not deter a woman from conceiving a child but might still impair pregnancy. If this mechanism had been at play, a job loss that happened in the pre-conception period would have been associated with a higher risk of non-live birth. Instead, we found no statistical association between a pre-conception job loss and adverse pregnancy outcomes. We interpret this finding as strengthening evidence on the importance of job loss during pregnancy, rather than job-related stressors experienced before conception, regarding adverse pregnancy outcomes.

The implications of our results are manifold. First, we contribute to a better understanding of the psycho-social factors influencing pregnancy loss, in this study job loss in particular, which are often hampered by poor-quality data sources and convenience samples (Gailey *etal.*, 2022a), through providing evidence using a longitudinal and nationally representative survey from the UK. We further suggest additional mechanisms associated with higher risk of pregnancy loss, such as stress and economic uncertainty following the exposure to a job loss during the pregnancy. Previous studies using survey or clinical data have identified plausible hormone-related (e.g. CRH) (Bruckner *etal.*, 2016) and behavioural (e.g. alcohol consumption, smoking) mechanisms (Margerison-Zilko *etal.*, 2017) that are associated with adverse pregnancy outcomes (Kramer *etal.*, 2000).

Second, our findings can be interpreted in the light of recent evidence from Denmark on the effect of partner’s job loss on other perinatal outcomes (Gailey *etal.*, 2022a). Exposure to partner’s job loss during the pregnancy leads to higher risk of low birthweight, while gestational length is unaffected. A recent meta-analysis also found that preterm birth is less responsive to maternal stressors than other pregnancy outcomes, such as birthweight (Lima *etal.*, 2018). If the risk of pregnancy loss is higher owing to external stressors, such as a job loss, and if the gestational age remains unaffected, this may induce selection *in utero*. In turn, it would lead to relatively better perinatal outcomes among the pregnancies that are carried to term (Margerison-Zilko *etal.*, 2017). However, this hypothesis must be confirmed by further evidence.

Our research is not free of limitations. First, we acknowledge the possibility of contextual self-selection into the risk of job loss during pregnancy and adverse pregnancy outcomes. For instance, a couple in which both partners suffer from physical or mental health issues might be more likely to experience a job loss and pregnancy disruption. Moreover, women from a less advantaged background might self-select into unions with partners of low SES who are, in turn, more prone to job precariousness (Gailey *etal.*, 2022a). The richness of the data allows us to control for a large set of confounders, which reduce this bias. Replication in larger samples, matching individual databases and firm registers might enable the use of matched-sibling (Gailey *etal.*, 2022a) or individual-fixed effect (Lindo, 2011) estimators to further reduce the bias if a job loss was not entirely independent of the birth outcome. In the UK, and in most European countries, individual-level data on pregnancy loss are very limited. Further, the integration with other sources of data providing insightful information on potential stressors is possible only in a few countries, such as Denmark (Gailey *etal.*, 2022a). The construction of such a ‘data infrastructure’ may contribute to systematically detecting the causality of stress-related risk factors on pregnancy outcomes.

Second, the availability of public registers would better discern clinically induced abortions, which tend to be under-reported in surveys (Desai *etal.*, 2021), and estimate the dates of conceptions with relevant clinical methods. Survey data are prone to under- or mis-reporting pregnancy experiences owing to the ambiguity of the pregnancy status and outcome (Bell and Fissell, 2021) and particularly in social contexts where abortion and pregnancy loss are socially stigmatized (Jones and Kost, 2007). Moreover, a miscarriage can be misreported as abortion, or vice versa, as these two terms were used interchangeably by the medical professionals up until the end of the 20^th^ century in the UK (Moscrop, 2013). The lack of consensus in the clinical language can create confusion in the way women communicate and report their early pregnancy experiences (Kolte *etal.*, 2015). We performed additional analyses using two alternative outcomes; miscarriage and non-live birth (a term encompassing miscarriage, stillbirth, and abortion) and the findings confirmed the initial operationalization of the outcome variable (Supplementary Tables S3 and S4).

At the same time, pregnancy recognition, therefore the accurate estimation of date of conception, depends on many social parameters including sexual and reproductive health knowledge (e.g. pregnancy symptoms, menstrual cycle tracking etc.), access to pregnancy testing and perceptions of one’s fecundity (Strong *etal.*, 2023). Moreover, having irregular menstrual cycles is an importance biological factor that could delay the pregnancy detection and confirmation (Nobles *etal.*, 2022). Hence, considering the complexity of pregnancy experiences, even the public registers or hospital records may fall short in accurately detecting pregnancies and estimating the date of conception, while the survey data may allow researchers to observe early miscarriages that did not take place in hospitals or clinics.

Lastly, even though the risk of exposure to disruptive life events, including job loss, and the coping mechanisms are socially stratified (Aquino *etal.*, 2022), the limited sample size of the survey data did not allow us to explore any heterogeneity across socio-economic groups. Lower-SES individuals, although more likely to experience disruptive events, such as job losses, may display stronger resilience towards economic precarity and vulnerability (Brand, 2015). Moreover, high-SES individuals may be more susceptible because job losses are relatively unexpected and non-normative events to them (Aquino *etal.*, 2022).

The strengths of our study include the identification of an exposure to a job loss during the pregnancy. In contrast to prior studies using self-reported job loss within 2 years before a live birth (Lindo, 2011; Scharber, 2014), we can detect if the exposure to a job loss occurred while the woman is pregnant, hence between the month of conception and the month of birth, or the latest month of pregnancy. This operationalization, unlike other measures of job separation, which do not distinguish between involuntary and anticipated causes of job end, reduces the bias related to unmeasured confounding by health and social factors that correlate with job loss. Prior research considered national or local unemployment rates as proxies for individual experiences of the economy. However, these measures could be subject to error (Margerison *etal.*, 2019). Further, if we consider the fact that some of the proposed mechanisms operate through stress, the unemployment rate—a proxy for economic distress—may not accurately capture this mechanism. A woman’s hardship during pregnancy may depend on her household’s economic circumstances more than on macroeconomic factors (Dehejia and Lleras-Muney, 2004).

From a global point of view, the replication of this analysis in different country contexts can assess the external validity of our findings and reveal to what extent the deployment of unemployment buffers cushions the consequences of job loss on gestations. The UK welfare state has an anti-poverty focus and provides low unemployment insurance benefits (Clasen and Clegg, 2011), whose replacement rates are relatively less generous than in the rest of Europe (34% of last job’s salary for 6 months on average). It is thus relevant to understand if social safety-net programmes in more generous welfare regimes more effectively redress the psycho-social hardship of job loss.

## Supplementary Material

dead183_Supplementary_Figure_S1

dead183_Supplementary_Figure_S2

dead183_Supplementary_Figure_S3

dead183_Supplementary_Table_S1

dead183_Supplementary_Table_S2

dead183_Supplementary_Table_S3

dead183_Supplementary_Table_S4

dead183_Supplementary_Table_S5

dead183_Supplementary_Table_S6

dead183_Supplementary_Table_S7

dead183_Supplementary_Table_S8

dead183_Supplementary_Table_S9

dead183_Supplementary_Table_S10

dead183_Supplementary_Table_S11

dead183_Supplementary_Table_S12

## Data Availability

The Institute for Social and Economic Research (ISER) at the University of Essex owns the copyright for Understanding Society (UKHLS) data used in this study. The UKHLS data are held/curated by the UK Data Service. Anyone wishing to use the UKHLS data (found at https://discover.ukdataservice.ac.uk/series/?sn1/42000031) must register and submit a data request to the UK Data Service at http://ukdataservice.ac.uk/. Additional terms and conditions of access are outlined at https://www.ukdataservice.ac.uk/get-data/how-to-access/conditions.
