# Supplementary figures and images for: Job loss during pregnancy and the risk of miscarriage and stillbirth
Source: Hum Reprod. 2023 Sep 27;38(11):2259–66. doi: 10.1093/humrep/dead183 (PMC10628490; doi:10.1093/humrep/dead183)

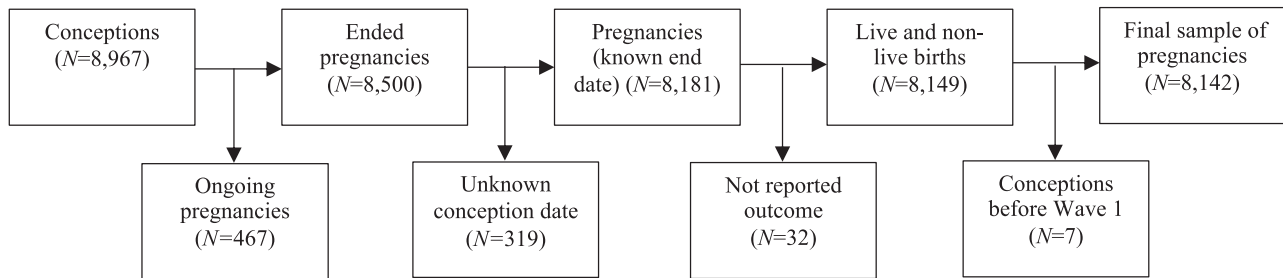

**Supplementary Figure S2.** Sample selection procedure.

Supplement: dead183_Supplementary_Figure_S2 [file dead183_supplementary_figure_s2.pdf]
