# Supplementary material for: Job loss during pregnancy and the risk of miscarriage and stillbirth
Source: Hum Reprod. 2023 Sep 27;38(11):2259–66. doi: 10.1093/humrep/dead183 (PMC10628490; doi:10.1093/humrep/dead183)
Supplement: dead183_Supplementary_Figure_S3 [file dead183_supplementary_figure_s3.pdf]

OXFORD

human reproduction

Human Reproduction, 2023, 1–1
https://doi.org/10.1093/humrep/dead183
Supplementary Data

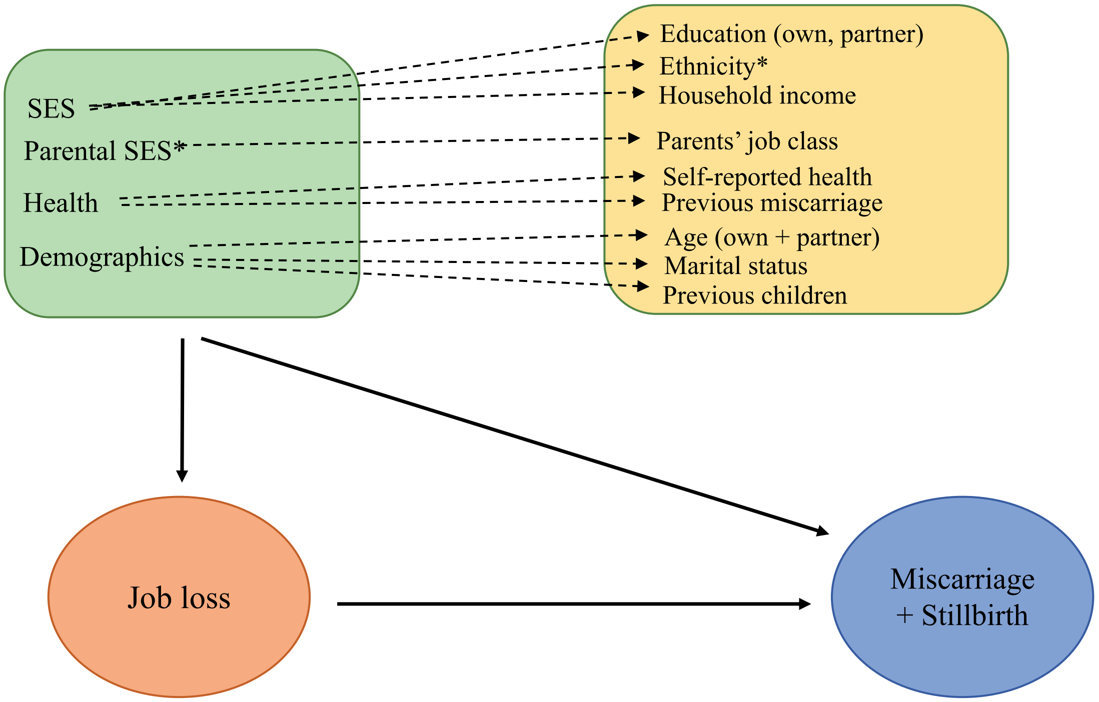

Supplementary Figure S3. Directed acyclic graph representing the relation between job loss and pregnancy loss. SES, socio-economic status. The green-filled shape represents the confounding factors while the yellow-filled shape displays the variables adjusted in the model to mitigate the effect of these confounding pathways. Dashed lines connect each confounding factor with the corresponding control variable, which is included in the model. The asterisk (*) denotes factors that remain constant across pregnancy observations.
