# Supplementary material for: Job loss during pregnancy and the risk of miscarriage and stillbirth
Source: Hum Reprod. 2023 Sep 27;38(11):2259–66. doi: 10.1093/humrep/dead183 (PMC10628490; doi:10.1093/humrep/dead183)
Supplement: dead183_Supplementary_Table_S1 [file dead183_supplementary_table_s1.pdf]

Human Reproduction, 2023, 1–1
https://doi.org/10.1093/humrep/dead183
Supplementary Data

Supplementary Table S1. Sample of data structure.

ID	#Preg.	Wave	Age	Ethnicity	Prior preg. loss	Union	Maternal status	Job loss during pregnancy	Partner's job class	Outcome
1	1	4	23–26	White British	No	Cohab.	Childless	No	Low-skilled	Live birth
2	1	4	31–34	Other	No	Married	Childless	No	Upper middle	Live birth
2	2	7	35–38	Other	No	Married	Mother	No	Upper middle	Loss
2	3	8	35–38	Other	Yes	Married	Mother	No	Missing	Live birth
3	1	2	27–30	Mixed	No	Single	Childless	No	Missing	Live birth
3	2	7	31–34	Mixed	No	Cohab.	Mother	Yes	Lower-middle	Live birth
4	1	3	39–41	White British	No	Married	Mother	No	Upper middle	Loss
4	2	4	39–41	White British	Yes	Married	Mother	No	Upper middle	Loss
5	1	9	19–22	Other Asian	No	Single	Childless	Yes	Missing	Live birth
6	1	10	35–38	White British	No	Cohab.	Childless	Yes	Low-skilled	Loss
6	2	11	39–42	White British	Yes	Married	Childless	No	Low-skilled	Live birth
7	1	5	39–41	Miss.	No	Cohab.	Mother	No	Upper middle	Live birth

Each line is an observation (pregnancy-woman).
