# Supplementary material for: Job loss during pregnancy and the risk of miscarriage and stillbirth
Source: Hum Reprod. 2023 Sep 27;38(11):2259–66. doi: 10.1093/humrep/dead183 (PMC10628490; doi:10.1093/humrep/dead183)
Supplement: dead183_Supplementary_Table_S2 [file dead183_supplementary_table_s2.pdf]

OXFORD
human reproduction
Human Reproduction, 2023, 1–1
https://doi.org/10.1093/humrep/dead183
Supplementary Data

Supplementary Table S2. Frequency of pregnancy duration (if prematurely ended) by the occurrence of job loss.

	No job loss	Job loss	Total
Less than 3 months	775	26	801
Between 3 and 6 months	156	6	162
6 months or more	18	1	19
	949	33	982
