# Supplementary material for: Job loss during pregnancy and the risk of miscarriage and stillbirth
Source: Hum Reprod. 2023 Sep 27;38(11):2259–66. doi: 10.1093/humrep/dead183 (PMC10628490; doi:10.1093/humrep/dead183)
Supplement: dead183_Supplementary_Table_S3 [file dead183_supplementary_table_s3.pdf]

OXFORD
human reproduction
Human Reproduction, 2023, 1–1
https://doi.org/10.1093/humrep/dead183
Supplementary Data

Supplementary Table S3. Frequency of pregnancy duration (if prematurely ended) by the reported cause of pregnancy loss.

	Miscarriage	Stillbirth	Total
Less than 3 months	801	0	801
Between 3 and 6 months	146	16	162
6 months or more	1	18	19
	948	34	982
