# Supplementary material for: Job loss during pregnancy and the risk of miscarriage and stillbirth
Source: Hum Reprod. 2023 Sep 27;38(11):2259–66. doi: 10.1093/humrep/dead183 (PMC10628490; doi:10.1093/humrep/dead183)
Supplement: dead183_Supplementary_Table_S4 [file dead183_supplementary_table_s4.pdf]

Human Reproduction, 2023, 1–1
https://doi.org/10.1093/humrep/dead183
Supplementary Data

Supplementary Table S4. Frequency of covariates by occurrence of job loss during pregnancy.

	Job loss	No job loss	Total
Age			
15–18	1	89	90
19–22	10	637	647
23–26	17	1155	1172
27–30	27	1766	1793
31–34	36	2087	2123
35–38	25	1472	1497
39–42	15	637	652
43–46	5	147	152
47–50	16	0	16
Ethnicity			
British/Irish	100	5806	5906
European/other White	7	333	340
Mixed: White and other	5	190	195
Indian	2	312	314
Pakistani	3	377	380
Bangladeshi	1	243	244
Other Asian/Asian British	3	140	143
Black/African/Caribbean/Black British	9	406	415
Other	3	64	67
Missing	3	135	138
Parents' social class			
Low-skilled working	15	1121	1136
Skilled working	23	1521	1544
Lower-middle	40	1648	1688
Upper-middle	34	2022	2056
Missing	24	1694	1718
Previous miscarriage			
None	121	7625	7746
1+ prior miscarriage	15	381	396
Presence of other children	41	2459	2500
Education			
Degree	62	2890	2952
Other higher	17	858	875
A level etc.	28	1714	1742
GCSE etc.	19	1546	1565
Other qualification	4	247	251
No qualification	4	329	333
Missing	2	422	424
Marital status			
Married	85	4530	4615
Cohabiting	32	1818	1850
Single	19	1658	1677
Partner's (if any) job class			
Semi-routine and routine	31	1142	1173
Intermediate	16	823	839
Management and professional	43	1960	2003
Missing	27	2423	2450
Inapplicable (no partner)	19	1658	1677

Notes: GCSE: General Certificate of Secondary Education; A-level: Advanced level. See Supplementary Table S5 for more details.
