# Supplementary material for: Job loss during pregnancy and the risk of miscarriage and stillbirth
Source: Hum Reprod. 2023 Sep 27;38(11):2259–66. doi: 10.1093/humrep/dead183 (PMC10628490; doi:10.1093/humrep/dead183)
Supplement: dead183_Supplementary_Table_S5 [file dead183_supplementary_table_s5.pdf]

OXFORD human reproduction

Human Reproduction, 2023, 1–1
https://doi.org/10.1093/humrep/dead183
Supplementary Data

Supplementary Table S5. List of variables.

Variable	Definition
Job loss (dichotomous)	Indicator of a job loss occurring because of a dismissal or redundancy between the month of conception and the month of reported pregnancy end.<br>A 'dismissal' is referred to a job separation caused by an individual worker's behaviour. A 'redundancy' refers to the total cessation of the employer's business (whether permanently or temporarily), cessation of business at the employee's workplace and reduction in the number of workers required to do a particular job, according to the British legislation.<br>*References*<br>Borland, J., Gregg, P., Knight, G., & Wadsworth, J. (1999). They get knocked down. Do they get up again? Displaced workers in Britain and Australia. In P. Kuhn (Ed.), *Losing work, moving on: International perspectives on worker displacement* (pp. 301–374). Kalamazoo: W. E. Upjohn Institute for Employment Research.<br>Mendolia, S. (2014) The impact of husband's job loss on partners' mental health. *Rev Econ Household* 12, 277–294. https://doi.org/10.1007/s11150-012-9149-6
Age (categorical)	4-year equally spaced age groups from '15–18' to '47–50'. In the analyses, the reference category is '27–30'
Ethnicity (categorical)	Ethnic group derived from multiple sources (self-reported as an adult, self-reported as a youth, reported by a household member, ethnic group of biological parents), with priority given to self-reported information.
Current job, Three Class NS-SEC (categorical)	Current job refers to being in paid employment during the last week, even if respondent was away from work in that week. It is based on the National Statistics Socio-economic Classification (NS-SEC). The job classification generally features 3, 5, or 8 macro-areas. The 3-category variable, which includes 'Low-skilled and working class', 'Intermediate', and 'Management & professional'. The NSSEC is coded post-field using information on type of employer, managerial duties and training. No code is assigned to those who are not currently employed. Details on the NS-SEC are provided by the Office for National Statistics (ONS), see http://www.ons.gov.uk/ons/guide-method/classifications/current-standard-classifications/soc2010/index.html
Parents' social class (categorical)	The higher of mother's and father's Standard Occupational Classification 2000/2010 (SOC2000/2010). It is based on a 4-digit version for Special Licence release only. The final output is a 4-group scheme in keeping with Oesch' classes (https://people.unil.ch/danieloesch/scripts/). The translation from the 4-digit version of UKHLS to the 4-group Oesch scheme ('low-skilled working'; 'skilled working'; 'lower-middle'; 'upper-middle') is based on Prof. Oesch script shared with the authors. If no information on parents' occupational status is reported, or if status is 'unemployed', the piece of information is coded as 'Missing'.<br>The classification is coded post-field using the respondent's description of their father's job. From Wave 3 onward, job descriptions were first coded to the code frame of SOC 2010 and the code for earlier versions of the classification was computed from a look-up file. For detailed information on the classification, see http://www.esds.ac.uk/doc/6411%5Cmrdoc%5Cpdf%5Csoc2000.pdf
Previous miscarriage (dichotomous)	Occurrence of a previous miscarriage during the surveyed period (Wave 1–12 of UKHLS).
Presence of other children (dichotomous)	Maternal status of the woman at the time of the conception
Education (categorical)	Highest educational level achieved over time. It is based on a derived variables harmonized by UKHLS. The categories are:<br>• 'Degree or higher': first degree or equivalent, MSc, MPhil, PhD, etc …<br>• 'Other higher qualification': diploma in higher education, nursing/other medical qualification.<br>• 'A level, etc … ' is a set of qualifications indicating the secondary school completion: A level, Welsh baccalaureate, international baccalaureate, AS level, Scottish Higher Grade, Certificate of sixth year studies).<br>• 'GCSE, etc … ': General Certificate of Secondary Education (GCSE) during Year 11 (students aged 15–16); O-Level (Ordinary Level)<br>• 'Other qualification': Certificate of Secondary Education (CSE), a secondary school qualification replaced by the GCSE; other school certifications.<br>• 'No qualification': none of the above.<br>The information is updated each year to include the most recent qualifications. The last highest qualification derived variable is fed forward and then updated with new qualifications obtained. Not all respondents were ever asked the highest educational qualification question. This group is mainly comprised of BHPS respondents. This group is coded as 'Missing'.
Self-reported health (categorical)	Self-assessed health (lagged): 'Excellent', 'Very good', 'Good', 'Fair', 'Poor', and 'Missing' (no information disclosed; no information from prior wave is available).
Marital status (categorical)	Single (not reporting the presence of a co-residential partner), cohabiting (reporting the presence of a non-married co-residential partner), married (reporting the presence of a married co-residential partner).

(continued)

Supplementary Table S5. (continued)

Variable	Definition
Partner's (if any) job class (categorical)	It is based on the National Statistics Socio-economic Classification (NS-SEC). The 3-category variable includes 'Routine', 'Intermediate', 'Management & professional', besides 'Missing' (no job reported), 'Inapplicable' (no partner reported).
Household income (continuous)	It results from the sum of the women and her partner's (if any) net income (labour earnings, dividends, transfers, etc …) during the month prior to the interview. It is lagged by one wave and expressed in natural logarithm British Pounds in the analyses.
Year and month fixed effects	Month (1–12) and year (2009–2022) of conception.
