# Supplementary material for: Job loss during pregnancy and the risk of miscarriage and stillbirth
Source: Hum Reprod. 2023 Sep 27;38(11):2259–66. doi: 10.1093/humrep/dead183 (PMC10628490; doi:10.1093/humrep/dead183)
Supplement: dead183_Supplementary_Table_S6 [file dead183_supplementary_table_s6.pdf]

Human Reproduction, 2023, 1–1
https://doi.org/10.1093/humrep/dead183
Supplementary Data

Supplementary Table S6. Logit model of pregnancy loss on job loss (extended version of **Table 2**).

	Model 1	Model 2	Model 3
Ref: no job loss			
A job loss	1.985***	1.831***	1.812***
	(0.414)	(0.379)	(0.378)
Age *(Ref: 27–30)*			
15–18	1.146	0.940	0.888
	(0.436)	(0.360)	(0.349)
19–22	1.218	1.068	1.054
	(0.194)	(0.180)	(0.182)
23–26	1.001	0.969	0.962
	(0.138)	(0.137)	(0.137)
31–34	1.222*	1.272**	1.274**
	(0.138)	(0.147)	(0.147)
35–38	1.530***	1.623***	1.632***
	(0.183)	(0.198)	(0.199)
39–42	2.294***	2.439***	2.449***
	(0.320)	(0.350)	(0.353)
43–46	4.819***	5.026***	5.076***
	(1.039)	(1.122)	(1.133)
47–50	6.886**	7.883**	7.933**
	(5.526)	(7.240)	(7.171)
Ethnicity *(Ref: White British)*			
European/other White	0.979	1.015	1.008
	(0.170)	(0.184)	(0.184)
Mixed: White and other	0.889	0.847	0.860
	(0.208)	(0.202)	(0.205)
Indian	0.849	0.808	0.809
	(0.168)	(0.170)	(0.171)
Pakistani	0.670**	0.645**	0.638**
	(0.137)	(0.137)	(0.137)
Bangladeshi	0.505**	0.468**	0.470**
	(0.151)	(0.142)	(0.142)
Other Asian/Asian British	0.793	0.686	0.683
	(0.230)	(0.198)	(0.198)
Black/African/Caribbean/Black British	0.854	0.830	0.834
	(0.141)	(0.142)	(0.144)
Other	0.986	0.971	0.961
	(0.363)	(0.383)	(0.386)
Missing	0.832	0.901	0.919
	(0.257)	(0.287)	(0.295)
Parents' highest class when woman was 16 yo *(Ref: low-skilled)*			
Skilled working	0.923	0.950	0.947
	(0.116)	(0.123)	(0.123)
Lower-middle	0.902	0.965	0.964
	(0.110)	(0.124)	(0.124)
Upper-middle	0.814*	0.889	0.892
	(0.100)	(0.116)	(0.117)
Missing	0.875	0.892	0.891
	(0.112)	(0.117)	(0.117)
Previous miscarriage *(Ref = none)*			
1+ prior miscarriage	12.706***	14.132***	14.205***
	(1.512)	(1.740)	(1.758)
Woman's highest qualification *(Ref: degree)*			
Other higher		1.014	0.991
		(0.129)	(0.127)
A level etc.		1.211*	1.187
		(0.129)	(0.128)
GCSE etc.		0.999	0.977
		(0.121)	(0.120)
Other qualification		0.899	0.888
		(0.219)	(0.216)
No qualification		1.344	1.341
		(0.264)	(0.266)
Missing		0.600*	0.617
		(0.181)	(0.191)

(continued)

Supplementary Table S6. (continued)

	Model 1	Model 2	Model 3
Partnership condition *(Ref: married)*			
Cohabiting		0.772**	0.787**
		(0.081)	(0.083)
Single		1.123	1.238*
		(0.126)	(0.159)
Maternal status *(Ref: childless)*			
Mother		0.482***	0.480***
		(0.046)	(0.046)
General health *(Ref: excellent)*			
Very good		0.827*	0.827*
		(0.087)	(0.087)
Good		1.031	1.028
		(0.114)	(0.114)
Fair		1.380**	1.376**
		(0.206)	(0.206)
Poor		1.421	1.429
		(0.384)	(0.387)
Current job, three class NS-SEC *(Ref: low-skilled and working class)*			
Intermediate			0.860
			(0.130)
Management and professional			0.873
			(0.108)
Missing			0.796*
			(0.096)
Income (ln)			0.978
			(0.024)
Missing income (ln)			0.798
			(0.157)
Year (*Ref*: 2009)			
2010	0.895	0.900	0.912
	(0.535)	(0.537)	(0.542)
2011	0.920	0.928	0.925
	(0.174)	(0.174)	(0.173)
2012	0.912	0.919	0.925
	(0.172)	(0.173)	(0.174)
2013	0.805	0.806	0.801
	(0.158)	(0.157)	(0.156)
2014	0.835	0.833	0.834
	(0.166)	(0.167)	(0.167)
2015	0.738	0.735	0.734
	(0.156)	(0.154)	(0.153)
2016	0.866	0.863	0.871
	(0.189)	(0.186)	(0.188)
2017	0.896	0.897	0.908
	(0.190)	(0.190)	(0.192)
2018	0.895	0.903	0.919
	(0.193)	(0.187)	(0.190)
2019	0.798	0.804	0.822
	(0.167)	(0.170)	(0.174)
2020	1.262	1.257	1.252
	(0.275)	(0.277)	(0.284)
2021	0.892	0.899	0.910
	(0.257)	(0.258)	(0.261)
2022	0.644	0.649	0.655
	(0.378)	(0.380)	(0.392)
Month (*Ref*: June)			
January	1.485*	1.500*	1.507*
	(0.272)	(0.280)	(0.284)
February	0.953	0.972	0.968
	(0.191)	(0.196)	(0.197)
March	1.258	1.275	1.272
	(0.241)	(0.249)	(0.245)
April	1.342	1.362	1.363
	(0.299)	(0.299)	(0.301)
May	1.222	1.215	1.207
	(0.239)	(0.239)	(0.240)
July	1.199	1.195	1.192
	(0.236)	(0.242)	(0.241)
August	1.330	1.229	1.226
	(0.241)	(0.240)	(0.239)
September	1.262	1.265	1.271
	(0.240)	(0.242)	(0.242)

(continued)

Supplementary Table S6. (continued)

	Model 1	Model 2	Model 3
October	1.402*	1.396*	1.391*
	(0.272)	(0.274)	(0.275)
November	1.364	1.361	1.362
	(0.271)	(0.269)	(0.270)
December	1.257	1.162	1.167
	(0.229)	(0.229)	(0.230)
Observations	8142	8142	8142

Notes: GCSE: General Certificate of Secondary Education; A-level: Advanced level; NS-SEC: National Statistics Socio-economic Classification. Odds ratios are estimated via logistic regression. SEs are in between parentheses.

*** $P < 0.01$.

** $P < 0.05$.

* $P < 0.1$.
