# Supplementary material for: Job loss during pregnancy and the risk of miscarriage and stillbirth
Source: Hum Reprod. 2023 Sep 27;38(11):2259–66. doi: 10.1093/humrep/dead183 (PMC10628490; doi:10.1093/humrep/dead183)
Supplement: dead183_Supplementary_Table_S7 [file dead183_supplementary_table_s7.pdf]

OXFORD
human reproduction

Human Reproduction, 2023, 1–1
https://doi.org/10.1093/humrep/dead183
Supplementary Data

Supplementary Table S7. Logit model of miscarriage on job loss.

	Model 1	Model 2	Model 3
Job loss	1.964***	1.799***	1.781***
	(0.411)	(0.372)	(0.371)
Age *(Ref: 27–30)*			
15–18	1.216	1.037	1.009
	(0.462)	(0.397)	(0.396)
19–22	1.130	1.012	1.007
	(0.187)	(0.177)	(0.181)
23–26	1.004	0.980	0.974
	(0.141)	(0.142)	(0.142)
31–34	1.249*	1.297**	1.299**
	(0.143)	(0.152)	(0.152)
35–38	1.558***	1.644***	1.652***
	(0.190)	(0.205)	(0.206)
39–42	2.346***	2.489***	2.500***
	(0.333)	(0.362)	(0.366)
43–46	4.791***	5.021***	5.066***
	(1.047)	(1.134)	(1.145)
47–50	7.395**	8.466**	8.546**
Ethnicity *(Ref: White British)*			
European/other White	1.004	1.049	1.045
	(0.177)	(0.193)	(0.192)
Mixed: White and other	0.880	0.839	0.851
	(0.211)	(0.204)	(0.208)
Indian	0.898	0.856	0.863
	(0.179)	(0.180)	(0.183)
Pakistani	0.600**	0.584**	0.581**
	(0.131)	(0.132)	(0.132)
Bangladeshi	0.535**	0.498**	0.503**
	(0.160)	(0.151)	(0.152)
Other Asian/Asian British	0.831	0.719	0.718
	(0.243)	(0.208)	(0.208)
Black/African/Caribbean/Black British	0.851	0.835	0.839
	(0.143)	(0.145)	(0.147)
Other	1.036	1.034	1.031
	(0.381)	(0.406)	(0.412)
Missing	0.880	0.956	0.973
	(0.274)	(0.307)	(0.315)
Parents' highest class when woman was 16 yo *(Ref: low-skilled)*			
Skilled working	0.928	0.947	0.944
	(0.120)	(0.125)	(0.125)
Lower-middle	0.933	0.984	0.982
	(0.116)	(0.129)	(0.129)
Upper-middle	0.819	0.879	0.882
	(0.103)	(0.117)	(0.118)
Missing	0.881	0.899	0.899
	(0.116)	(0.121)	(0.120)
Previous miscarriage *(Ref: none)*			
1+ prior miscarriage	13.266***	14.602***	14.673***
	(1.584)	(1.804)	(1.823)
Woman's highest qualification *(Ref: degree)*			
Other higher		1.032	1.008
		(0.133)	(0.131)
A level, etc.		1.216*	1.190
		(0.132)	(0.130)
GCSE, etc.		0.962	0.938
		(0.119)	(0.118)
Other qualification		0.891	0.877
		(0.220)	(0.218)
No qualification		1.257	1.251
		(0.253)	(0.254)
Missing		0.531**	0.543*
		(0.166)	(0.176)
Partnership condition *(Ref: married)*			
Cohabiting		0.770**	0.783**
		(0.082)	(0.085)

(continued)

Supplementary Table S7. (continued)

	Model 1	Model 2	Model 3
Single		1.092	1.199
		(0.126)	(0.158)
Maternal status *(Ref: childless)*			
Mother		0.492***	0.490***
		(0.048)	(0.048)
General health *(Ref: excellent)*			
Very good		0.837*	0.836*
		(0.090)	(0.090)
Good		1.021	1.017
		(0.116)	(0.115)
Fair		1.343*	1.336*
		(0.204)	(0.204)
Poor		1.441	1.446
		(0.396)	(0.398)
Current job, three class NS-SEC *(Ref: low-skilled and working class)*			
Intermediate			0.832
			(0.128)
Management and professional			0.849
			(0.106)
Missing			0.783**
			(0.096)
Income (ln)			0.985
			(0.025)
Missing income (ln)			0.809
			(0.162)
Year and month FE	Yes	Yes	Yes
Observations	8142	8142	8142

Notes: GCSE: General Certificate of Secondary Education; A-level: Advanced level; NS-SEC: National Statistics Socio-economic Classification. Odds ratios are estimated via logistic regression. SEs are in between parentheses.

*** $P < 0.01$.

** $P < 0.05$.

* $P < 0.1$.
