# Supplementary material for: Job loss during pregnancy and the risk of miscarriage and stillbirth
Source: Hum Reprod. 2023 Sep 27;38(11):2259–66. doi: 10.1093/humrep/dead183 (PMC10628490; doi:10.1093/humrep/dead183)
Supplement: dead183_Supplementary_Table_S8 [file dead183_supplementary_table_s8.pdf]

OXFORD
human reproduction

Human Reproduction, 2023, 1–1
https://doi.org/10.1093/humrep/dead183
Supplementary Data

Supplementary Table S8. Logit model of non-live birth on job loss.

	Model 1	Model 2	Model 3
Job loss	1.762***	1.667**	1.660**
	(0.361)	(0.338)	(0.340)
Age *(Ref: 27–30)*			
15–18	2.105**	1.374	1.336
	(0.622)	(0.411)	(0.419)
19–22	1.929***	1.409**	1.401**
	(0.262)	(0.205)	(0.208)
23–26	1.322**	1.178	1.172
	(0.161)	(0.150)	(0.150)
31–34	1.194*	1.269**	1.273**
	(0.127)	(0.139)	(0.139)
35–38	1.500***	1.622***	1.630***
	(0.169)	(0.187)	(0.188)
39–42	2.377***	2.603***	2.599***
	(0.313)	(0.355)	(0.356)
43–46	5.185***	5.428***	5.456***
	(1.073)	(1.169)	(1.175)
47–50	6.309**	6.899**	6.878**
	(4.961)	(6.141)	(6.048)
Ethnicity *(Ref: White British)*			
European/other White	1.011	1.110	1.100
	(0.163)	(0.187)	(0.187)
Mixed: White and other	0.919	0.816	0.821
	(0.200)	(0.177)	(0.178)
Indian	0.876	0.921	0.941
	(0.158)	(0.178)	(0.182)
Pakistani	0.511***	0.547***	0.546***
	(0.105)	(0.117)	(0.117)
Bangladeshi	0.439***	0.439***	0.446***
	(0.122)	(0.125)	(0.127)
Other Asian/Asian British	0.832	0.734	0.734
	(0.217)	(0.197)	(0.197)
Black/African/Caribbean/Black British	0.840	0.740*	0.742*
	(0.128)	(0.117)	(0.119)
Other	0.952	0.992	0.997
	(0.332)	(0.370)	(0.379)
Missing	0.942	1.013	1.027
	(0.246)	(0.270)	(0.276)
Parents' highest class when woman was 16 yo *(Ref: low-skilled)*			
Skilled working	0.886	0.917	0.912
	(0.104)	(0.113)	(0.112)
Lower-middle	0.918	1.001	1.002
	(0.105)	(0.122)	(0.123)
Upper-middle	0.821*	0.921	0.925
	(0.094)	(0.114)	(0.115)
Missing	0.990	0.988	0.986
	(0.116)	(0.119)	(0.119)
Previous miscarriage *(Ref: none)*			
1+ prior miscarriage	10.451***	11.807***	11.968***
	(1.237)	(1.454)	(1.482)
Woman's highest qualification *(Ref: degree)*			
Other higher		0.960	0.933
		(0.116)	(0.113)
A level, etc.		1.250**	1.217*
		(0.125)	(0.123)
GCSE, etc.		0.908	0.882
		(0.104)	(0.103)
Other qualification		0.825	0.811
		(0.186)	(0.184)
No qualification		1.144	1.137
		(0.212)	(0.213)
Missing		0.515**	0.581*
		(0.142)	(0.168)

(continued)

Supplementary Table S8. (continued)

	**Model 1**	**Model 2**	**Model 3**
Partnership condition *(Ref: married)*			
Cohabiting		0.823**	0.833*
		(0.081)	(0.083)
Single		1.830***	1.984***
		(0.184)	(0.230)
Maternal status *(Ref: childless)*			
Mother		0.481***	0.480***
		(0.042)	(0.042)
General health *(Ref: excellent)*			
Very good		0.877	0.876
		(0.086)	(0.086)
Good		1.008	1.004
		(0.105)	(0.104)
Fair		1.422**	1.412**
		(0.196)	(0.196)
Poor		1.508	1.504
		(0.380)	(0.383)
Current job, three class NS-SEC *(Ref: low-skilled and working class)*			
Intermediate			0.826
			(0.121)
Management and professional			0.848
			(0.100)
Missing			0.797**
			(0.092)
Income (ln)			0.981
			(0.023)
Missing income (ln)			0.653**
			(0.126)
Year and month FE	Yes	Yes	Yes
Observations	8142	8142	8142

Notes: GCSE: General Certificate of Secondary Education; A-level: Advanced level; NS-SEC: National Statistics Socio-economic Classification. Odds ratios are estimated via logistic regression. SEs are in between parentheses.

*** $P < 0.01$.

** $P < 0.05$.

* $P < 0.1$.
