# Supplementary material for: Job loss during pregnancy and the risk of miscarriage and stillbirth
Source: Hum Reprod. 2023 Sep 27;38(11):2259–66. doi: 10.1093/humrep/dead183 (PMC10628490; doi:10.1093/humrep/dead183)
Supplement: dead183_Supplementary_Table_S9 [file dead183_supplementary_table_s9.pdf]

OXFORD human reproduction

Human Reproduction, 2023, 1–1
https://doi.org/10.1093/humrep/dead183
Supplementary Data

Supplementary Table S9. Logit model of pregnancy loss on different instances of job end (involuntary job loss, anticipated contract end, other).

	Model 1	Model 2	Model 3
Job end *(Ref = none)*			
Involuntary job loss	2.013***	1.858***	1.839***
	(0.422)	(0.387)	(0.386)
Contract end	1.821	1.644	1.621
	(0.798)	(0.790)	(0.793)
Other	0.755	0.723	0.710
	(0.246)	(0.232)	(0.227)
Age *(Ref: 27–30)*			
15–18	1.159	0.949	0.896
	(0.441)	(0.364)	(0.353)
19–22	1.229	1.079	1.065
	(0.196)	(0.182)	(0.184)
23–26	1.003	0.971	0.964
	(0.138)	(0.138)	(0.137)
31–34	1.225*	1.275**	1.278**
	(0.138)	(0.147)	(0.147)
35–38	1.533***	1.626***	1.635***
	(0.184)	(0.199)	(0.200)
39–42	2.291***	2.434***	2.445***
	(0.319)	(0.349)	(0.352)
43–46	4.810***	5.015***	5.066***
	(1.035)	(1.118)	(1.130)
47–50	6.890**	7.884**	7.935**
	(5.538)	(7.251)	(7.183)
Ethnicity *(Ref: White British)*			
European/other White	0.978	1.013	1.006
	(0.170)	(0.185)	(0.184)
Mixed: White and other	0.887	0.845	0.858
	(0.208)	(0.201)	(0.205)
Indian	0.852	0.812	0.812
	(0.169)	(0.171)	(0.172)
Pakistani	0.670**	0.646**	0.639**
	(0.137)	(0.138)	(0.137)
Bangladeshi	0.508**	0.470**	0.471**
	(0.152)	(0.142)	(0.143)
Other Asian/Asian British	0.794	0.686	0.683
	(0.231)	(0.198)	(0.198)
Black/African/Caribbean/Black British	0.856	0.831	0.836
	(0.142)	(0.142)	(0.144)
Other	0.986	0.969	0.958
	(0.363)	(0.382)	(0.385)
Missing	0.830	0.898	0.915
	(0.257)	(0.286)	(0.294)
Parents' highest class when woman was 16 yo *(Ref: low-skilled)*			
Skilled working	0.923	0.950	0.947
	(0.116)	(0.123)	(0.123)
Lower-middle	0.904	0.967	0.965
	(0.110)	(0.124)	(0.124)
Upper-middle	0.816*	0.891	0.895
	(0.100)	(0.117)	(0.117)
Missing	0.873	0.892	0.891
	(0.112)	(0.117)	(0.117)
Previous miscarriage *(Ref: none)*			
1+ prior miscarriage	12.767***	14.200***	14.277***
	(1.521)	(1.752)	(1.771)
Woman's highest qualification *(Ref: degree)*			
Other higher		1.014	0.991
		(0.129)	(0.127)
A level, etc.		1.214*	1.189
		(0.129)	(0.128)
GCSE, etc.		0.997	0.974
		(0.121)	(0.120)

(continued)

Supplementary Table S9. (continued)

	Model 1	Model 2	Model 3
Other qualification		0.898	0.887
		(0.218)	(0.216)
No qualification		1.350	1.346
		(0.265)	(0.267)
Missing		0.602*	0.618
		(0.181)	(0.192)
Partnership condition *(Ref: married)*			
Cohabiting		0.773**	0.787**
		(0.081)	(0.083)
Single		1.123	1.238*
		(0.127)	(0.159)
Maternal status *(Ref: childless)*			
Mother		0.483***	0.481***
		(0.046)	(0.046)
General health *(Ref: excellent)*			
Very good		0.826*	0.826*
		(0.087)	(0.087)
Good		1.029	1.025
		(0.114)	(0.114)
Fair		1.376**	1.372**
		(0.205)	(0.205)
Poor		1.414	1.421
		(0.384)	(0.387)
Current job, three class NS-SEC *(Ref: low-skilled and working class)*			
Intermediate			0.859
			(0.130)
Management and professional			0.871
			(0.108)
Missing			0.795*
			(0.096)
Income (ln)			0.977
			(0.024)
Missing income (ln)			0.797
			(0.156)
Year and month FE	Yes	Yes	Yes
Observations	8142	8142	8142

Notes: GCSE: General Certificate of Secondary Education; A-level: Advanced level; NS-SEC: National Statistics Socio-economic Classification. Odds ratios are estimated via logistic regression. SEs are in between parentheses.

*** $P < 0.01$.

** $P < 0.05$.

* $P < 0.1$.
