# Supplementary material for: Job loss during pregnancy and the risk of miscarriage and stillbirth
Source: Hum Reprod. 2023 Sep 27;38(11):2259–66. doi: 10.1093/humrep/dead183 (PMC10628490; doi:10.1093/humrep/dead183)
Supplement: dead183_Supplementary_Table_S10 [file dead183_supplementary_table_s10.pdf]

OXFORD
human reproduction
Human Reproduction, 2023, 1–1
https://doi.org/10.1093/humrep/dead183
Supplementary Data

Supplementary Table S10. Logit model of pregnancy loss on the timing of job loss (if any) with respect to conception.

	Model 1	Model 2	Model 3
Ref: a job loss *preceding* conception by 12–1 month			
In utero exposure	2.429**	2.298**	2.277**
	(0.984)	(0.931)	(0.923)
Job loss between the gestation end and 12 months thereafter	1.555	1.477	1.440
	(0.646)	(0.615)	(0.602)
Job loss in another period, or no job loss	1.232	1.293	1.298
	(0.434)	(0.452)	(0.454)
Age *(Ref: 27–30)*			
15–18	1.151	0.944	0.887
	(0.438)	(0.362)	(0.348)
19–22	1.213	1.064	1.048
	(0.193)	(0.179)	(0.181)
23–26	0.997	0.969	0.961
	(0.138)	(0.138)	(0.137)
31–34	1.220*	1.270**	1.273**
	(0.137)	(0.146)	(0.147)
35–38	1.528***	1.622***	1.632***
	(0.183)	(0.198)	(0.199)
39–42	2.278***	2.425***	2.438***
	(0.318)	(0.348)	(0.351)
43–46	4.828***	5.040***	5.089***
	(1.040)	(1.122)	(1.133)
47–50	6.218**	7.133**	7.198**
	(4.999)	(6.548)	(6.505)
Ethnicity *(Ref: White British)*			
European/other White	0.979	1.013	1.006
	(0.171)	(0.184)	(0.184)
Mixed: White and other	0.890	0.846	0.859
	(0.208)	(0.202)	(0.205)
Indian	0.840	0.799	0.799
	(0.167)	(0.169)	(0.170)
Pakistani	0.672*	0.645**	0.638**
	(0.137)	(0.138)	(0.136)
Bangladeshi	0.500**	0.465**	0.466**
	(0.149)	(0.141)	(0.141)
Other Asian/Asian British	0.799	0.688	0.684
	(0.233)	(0.200)	(0.199)
Black/African/Caribbean/Black British	0.865	0.839	0.843
	(0.143)	(0.143)	(0.145)
Other	0.995	0.977	0.965
	(0.366)	(0.386)	(0.388)
Missing	0.829	0.898	0.916
	(0.256)	(0.286)	(0.294)
Parents' highest class when woman was 16 yo *(Ref: low-skilled)*			
Skilled working	0.926	0.953	0.950
	(0.117)	(0.124)	(0.123)
Lower-middle	0.906	0.968	0.967
	(0.111)	(0.125)	(0.125)
Upper-middle	0.818	0.892	0.895
	(0.100)	(0.117)	(0.117)
Missing	0.877	0.893	0.892
	(0.113)	(0.117)	(0.117)
Previous miscarriage *(Ref: none)*			
1+ prior miscarriage	12.751***	14.194***	14.268***
	(1.514)	(1.744)	(1.763)
Woman's highest qualification *(Ref: degree)*			
Other higher		1.019	0.996
		(0.130)	(0.128)
A level, etc.		1.211*	1.187
		(0.129)	(0.127)
GCSE, etc.		1.001	0.978
		(0.121)	(0.121)
Other qualification		0.903	0.892
		(0.219)	(0.217)

(continued)

Supplementary Table S10. (continued)

	Model 1	Model 2	Model 3
No qualification		1.348	1.343
		(0.265)	(0.267)
Missing		0.602*	0.618
		(0.181)	(0.192)
Partnership condition *(Ref: married)*			
Cohabiting		0.769**	0.783**
		(0.080)	(0.083)
Single		1.121	1.233
		(0.127)	(0.159)
Maternal status *(Ref: childless)*			
Mother		0.483***	0.481***
		(0.046)	(0.046)
General health *(Ref: excellent)*			
Very good		0.828*	0.828*
		(0.087)	(0.087)
Good		1.032	1.028
		(0.114)	(0.114)
Fair		1.378**	1.376**
		(0.206)	(0.206)
Poor		1.422	1.430
		(0.384)	(0.388)
Current job, three class NS-SEC *(Ref: low-skilled and working class)*			
Intermediate			0.864
			(0.131)
Management and professional			0.876
			(0.108)
Not specified			0.800*
			(0.097)
Income (ln)			0.976
			(0.024)
Missing income (ln)			0.802
			(0.157)
Month and year FE	Yes	Yes	Yes
Observations	8142	8142	8142

Notes: GCSE: General Certificate of Secondary Education; A-level: Advanced level; NS-SEC: National Statistics Socio-economic Classification. Odds ratios are estimated via logistic regression. Ses are in between parentheses.

*** $P < 0.01$.

** $P < 0.05$.

* $P < 0.1$.
