# Supplementary material for: Job loss during pregnancy and the risk of miscarriage and stillbirth
Source: Hum Reprod. 2023 Sep 27;38(11):2259–66. doi: 10.1093/humrep/dead183 (PMC10628490; doi:10.1093/humrep/dead183)
Supplement: dead183_Supplementary_Table_S11 [file dead183_supplementary_table_s11.pdf]

Human Reproduction, 2023, 1–1
https://doi.org/10.1093/humrep/dead183
Supplementary Data

Supplementary Table S11. Logit model of pregnancy loss on woman and partner's job loss.

	Model 1	Model 2	Model 3
Ref: no job loss			
Woman's job loss	1.973**	1.734**	1.748**
	(0.552)	(0.484)	(0.491)
Partner's job loss	1.978**	1.937**	1.933**
	(0.602)	(0.587)	(0.586)
		Woman's job loss = partner's job loss	
	$\chi^2(1) = 0.03$	$\chi^2(1) = 0.12$	$\chi^2(1) = 0.11$
	$P > \chi^2 = 0.902$	$P > \chi^2 = 0.764$	$P > \chi^2 = 0.769$
Age *(Ref: 27–30)*			
15–18	1.144	0.913	0.851
	(0.430)	(0.348)	(0.333)
19–22	1.219	1.045	1.023
	(0.190)	(0.174)	(0.174)
23–26	1.004	0.960	0.953
	(0.137)	(0.136)	(0.135)
31–34	1.212*	1.270**	1.275**
	(0.137)	(0.147)	(0.147)
35–38	1.514***	1.620***	1.628***
	(0.181)	(0.198)	(0.198)
39–42	2.274***	2.440***	2.435***
	(0.318)	(0.350)	(0.352)
43–46	4.758***	5.007***	5.017***
	(1.026)	(1.118)	(1.119)
47–50	6.856**	7.867**	7.772**
	(5.641)	(7.286)	(7.112)
Ethnicity *(Ref: White British)*			
European/other White	0.974	1.015	1.006
	(0.170)	(0.185)	(0.184)
Mixed: White and other	0.884	0.844	0.844
	(0.207)	(0.201)	(0.200)
Indian	0.843	0.805	0.801
	(0.168)	(0.169)	(0.170)
Pakistani	0.684*	0.647**	0.640**
	(0.138)	(0.137)	(0.135)
Bangladeshi	0.510**	0.464**	0.461***
	(0.150)	(0.139)	(0.138)
Other Asian/Asian British	0.795	0.686	0.682
	(0.228)	(0.196)	(0.195)
Black/African/Caribbean/Black British	0.851	0.825	0.824
	(0.141)	(0.141)	(0.141)
Other	0.981	0.975	0.966
	(0.360)	(0.384)	(0.382)
Missing	0.819	0.881	0.886
	(0.250)	(0.276)	(0.278)
Parents' highest class when woman was 16 yo *(Ref: low-skilled)*			
Skilled working	0.923	0.949	0.948
	(0.116)	(0.123)	(0.123)
Lower-middle	0.902	0.965	0.967
	(0.110)	(0.124)	(0.124)
Upper-middle	0.814*	0.888	0.890
	(0.100)	(0.116)	(0.117)
Missing	0.875	0.892	0.889
	(0.112)	(0.117)	(0.116)
Previous miscarriage *(Ref: none)*			
1+ prior miscarriage	12.916***	14.270***	14.364***
	(1.534)	(1.750)	(1.765)
Woman's highest qualification *(Ref: degree)*			
Other higher		1.025	1.014
		(0.130)	(0.129)
A level etc.		1.231**	1.220*
		(0.128)	(0.127)
GCSE etc.		1.013	1.002
		(0.120)	(0.119)

(continued)

Supplementary Table S11. (continued)

	Model 1	Model 2	Model 3
Other qualification		0.913	0.911
		(0.221)	(0.220)
No qualification		1.377*	1.365
		(0.266)	(0.265)
Missing		0.601*	0.640
		(0.180)	(0.197)
Partnership condition *(Ref: married)*			
Cohabiting		0.773**	0.774**
		(0.081)	(0.081)
Single		1.124	1.132
		(0.126)	(0.127)
Maternal status *(Ref: childless)*			
Mother		0.481***	0.481***
		(0.046)	(0.046)
General health *(Ref: excellent)*			
Very good		0.826*	0.828*
		(0.087)	(0.087)
Good		1.031	1.030
		(0.114)	(0.114)
Fair		1.384**	1.382**
		(0.206)	(0.206)
Poor		1.418	1.408
		(0.384)	(0.382)
Current job, three class NS-SEC *(Ref: low-skilled and working class)*			
Intermediate			0.795*
			(0.096)
Management and professional			0.858
			(0.130)
Not specified			0.873
			(0.108)
Income (ln)			0.978
			(0.024)
Missing income (ln)			0.799
			(0.157)
Month and year FE	Yes	Yes	Yes
Observations	8142	8142	8142

Notes: GCSE: General Certificate of Secondary Education; A-level: Advanced level; NS-SEC: National Statistics Socio-economic Classification. Odds ratios are estimated via logistic regression. SEs are in between parentheses.

*** $P < 0.01$.

** $P < 0.05$.

* $P < 0.1$.
