# Supplementary material for: Job loss during pregnancy and the risk of miscarriage and stillbirth
Source: Hum Reprod. 2023 Sep 27;38(11):2259–66. doi: 10.1093/humrep/dead183 (PMC10628490; doi:10.1093/humrep/dead183)
Supplement: dead183_Supplementary_Table_S12 [file dead183_supplementary_table_s12.pdf]

OXFORD
human reproduction
Human Reproduction, 2023, 1–1
https://doi.org/10.1093/humrep/dead183
Supplementary Data

Supplementary Table S12. Logit model of pregnancy loss on job loss (without controlling for previous miscarriages).

	Model 1	Model 2	Model 3
Ref: no job loss			
A job loss	2.251***	2.079***	2.034***
	(0.465)	(0.438)	(0.432)
Age *(Ref: 27–30)*			
15–18	1.020	0.917	0.852
	(0.392)	(0.358)	(0.338)
19–22	1.150	1.050	1.034
	(0.182)	(0.175)	(0.176)
23–26	0.929	0.912	0.905
	(0.126)	(0.125)	(0.125)
31–34	1.277**	1.312**	1.322**
	(0.138)	(0.145)	(0.146)
35–38	1.637***	1.677***	1.699***
	(0.185)	(0.194)	(0.196)
39–42	2.660***	2.782***	2.834***
	(0.340)	(0.366)	(0.374)
43–46	5.868***	6.322***	6.419***
	(1.107)	(1.240)	(1.257)
47–50	12.298***	14.413***	14.881***
	(6.701)	(8.216)	(8.415)
Ethnicity *(Ref: White British)*			
European/other White	0.942	1.036	1.031
	(0.155)	(0.176)	(0.176)
Mixed: White and other	0.839	0.824	0.837
	(0.198)	(0.195)	(0.199)
Indian	0.812	0.812	0.800
	(0.152)	(0.157)	(0.155)
Pakistani	0.500***	0.499***	0.490***
	(0.104)	(0.107)	(0.105)
Bangladeshi	0.423***	0.407***	0.401***
	(0.124)	(0.122)	(0.120)
Other Asian/Asian British	0.818	0.722	0.712
	(0.208)	(0.190)	(0.188)
Black/African/Caribbean/Black British	0.743*	0.748*	0.744*
	(0.123)	(0.126)	(0.126)
Other	0.906	0.930	0.907
	(0.329)	(0.365)	(0.362)
Missing	1.114	1.219	1.238
	(0.325)	(0.368)	(0.376)
Parents' highest class when woman was 16 yo *(Ref: low-skilled)*			
Skilled working	0.825	0.833	0.835
	(0.099)	(0.102)	(0.102)
Lower-middle	0.823*	0.861	0.862
	(0.095)	(0.104)	(0.104)
Upper-middle	0.692***	0.744**	0.752**
	(0.080)	(0.091)	(0.092)
Missing	0.758**	0.754**	0.756**
	(0.094)	(0.096)	(0.096)
Woman's highest qualification *(Ref: degree)*			
Other higher		1.074	1.048
		(0.129)	(0.127)
A level etc.		1.367***	1.335***
		(0.136)	(0.134)
GCSE etc.		0.987	0.960
		(0.114)	(0.113)
Other qualification		0.744	0.727
		(0.180)	(0.176)
No qualification		1.205	1.186
		(0.234)	(0.233)
Missing		0.642	0.609
		(0.190)	(0.184)
Partnership condition *(Ref: married)*			
Cohabiting		0.769***	0.780**
		(0.076)	(0.078)

(continued)

Supplementary Table S12. (continued)

	Model 1	Model 2	Model 3
Single		1.075	1.181
		(0.117)	(0.147)
Maternal status *(Ref: childless)*			
Mother		0.651***	0.647***
		(0.054)	(0.054)
General health *(Ref: excellent)*			
Very good		0.838*	0.837*
		(0.084)	(0.084)
Good		1.046	1.040
		(0.110)	(0.109)
Fair		1.349**	1.342**
		(0.194)	(0.193)
Poor		1.803**	1.803**
		(0.434)	(0.435)
Current job, three class NS-SEC *(Ref: low-skilled and working class)*			
Intermediate			0.816
			(0.113)
Management and professional			0.832
			(0.096)
Not specified			0.779**
			(0.088)
Income (ln)			0.976
			(0.023)
Missing income (ln)			0.977
			(0.184)
Month and year FE	Yes	Yes	Yes
Observations	8142	8142	8142

Notes: GCSE: General Certificate of Secondary Education; A-level: Advanced level; NS-SEC: National Statistics Socio-economic Classification. Odds ratios are estimated via logistic regression. SEs are in between parentheses.

*** $P < 0.01$.

** $P < 0.05$.

* $P < 0.1$.
